# Bogus Medical Colleges

**Published:** 1891-12

**Authors:** 


### EDITORIAL.

### Bogus Medical College.

A revelation has been made within the last few weeks through the energy and perseverance of the *Cincinnati Commercial-Gazette*, that has created great surprise in the mind of the public not only in Cincinnati, but throughout the entire country. It is nothing less than a bogus medical college, run in the interest of one Dr. Van Vleck. This concern seems to have been organized and conducted for the purpose of issuing and selling medical diplomas, and it is said dental diplomas as well; so that not only the medical profession but the dental as well is interested in this expose.

Since attention has been called to this matter the medical profession of the country has been aroused to an unusual degree, and medical schools as well, and all are now enquiring what shall be done, not only in reference to this case, but how shall such institutions be prevented in the future. This institute was conducted much upon the same method as one discovered in Philadelphia some years ago, conducted by a Dr. Buchannon, which, as well as this, issued dental diplomas. Medical societies have had this subject under discussion during the last two or three weeks, considering and devising methods of relief from such enterprises as this.

It is a most alarming state of affairs when men with no medical knowledge are authorized to go abroad in communities and ply the vocation of a physician and surgeon with blank ignorance, and more likely to kill than to cure. Not only have medical societies taken up this matter, but the medical colleges also. On the 2nd inst. a meeting of the representatives of the various medical colleges of Ohio was held in Columbus, and after considering the whole ground, the conclusion arrived at, was that relief can only be had in stringent legislation. A committee was therefore, appointed to formulate a bill to be submitted to the legislature for this purpose, and it is to be hoped it will be wisely constructed and sufficient to accomplish the desired end. This effort will doubtless have the support and co-operation of every medical society in the State, and undoubtedly the moral support of similar societies in the neighboring States, and from medical colleges as well. The bill, though not yet completely prepared, will embrace the best features of the laws of the States which have taken the foremost position as to medical legislation; it provides for the establishment of a State Examining Board of seven members to be appointed by the governor, one member retiring each year; the members of the board to receive $5.00 per day and expenses for the time actually employed in the transaction of business. The board will examine all new graduates in medicine, and those desiring to practice without a diploma. The board can, at its discretion, accept the diplomas of graduates of medical colleges when it is satisfied with the attainments of the applicant. The bill further provides that each diploma must be accompanied by an affidavit to the effect that the person presenting it is the one to whom it was issued; and the presentation of a fraudulent diploma is made a crime under the Forgery Act and punished accordingly. Each certificate must be recorded in the county in which the holder lives. The bill will further provide that itinerant vendors of drugs or medicines, or those who advertise proposals to cure or treat disease or deformity, with any special drug, shall pay each to the treasurer of the board the sum of $100 per month, and this in addition to passing a satisfactory examination. The physician who has practiced medicine or surgery for ten years previous to the passage of this bill will be held to have fulfilled the requirements of the law. The penalty for violation of the law will be a fine in from $100 to $200.

The various schools of medicine, as the Allopathic, the Homœpathic and the Eclectic have joined issue in the procurement of this law, which is a very gratifying thing indeed, and one prophetic of more certain success than has been reached hitherto. The schisms in the medical ranks have stood squarely in the way of procuring adequate legislation hitherto; now it seems success must attend this effort, and not only are medical men and those in the practice of dentistry as well interested in this matter, but dental colleges are interested as well as medical colleges and dental societies also.

At a recent meeting of the Odontological Society of Cincinnati, after a free and full consideration of the subject, the following resolutions were unanimously adopted:

"*Resolved*, That in view of the recent disclosures by the *Cincinnati Commercial-Gazette* of the fraudulent issue of medical and dental diplomas by the so-called Medical University of Ohio, we regard the present an opportune time for the dental profession of this city to take measures for obtaining an efficient law for regulating the practice of dentistry in Ohio."

"*Resolved*, That the Cincinnati Odontological Society shall arrange to co-operate with the Ohio State Dental Society, and other dental societies of the State, in asking the legislature to pass the bill known as an 'Act to Regulate the Practice of Dentistry in the State of Ohio,' which was introduced last winter."

After which, the unanimous opinion of the society was expressed that in this matter the *Commercial-Gazette* had exhibited the true journalistic enterprise, and that its managers are entitled to the thanks of the dental profession in this city, and of Ohio.

